# On the Treatment of Delirium Tremens

**Published:** 1855-01

**Authors:** Alexander Peddie

**Affiliations:** Edinburgh


### ON THE TREATMENT OF DELIRIUM TREMENS.

BY ALEXANDER PEDDIE, M. D., EDINBURGH.

As regards the treatment of delirium tremens on the views which I have endeavored to unfold, I may plead the experience of upwards of fifteen years; and state, that during five previous years I also had ample opportunities of witnessing the practice of others, and of personally testing the merits of the mode of treatment then, and still ordinarily pursued. In the earlier period of practice the observations were made almost entirely in connection with hospital and dispensary attendance, affording a great many examples of the disease in its pure and in its complicated forms, as occurring among tavern keepers, brewers, butchers, and the lowest order of dram-drinkers generally; latterly the instances have been mostly among a better class of society, yet the disease presenting the same features, and originating from the same degrading cause.—The frequent sudden fatalities which I witnessed from arachnitis, convulsions and coma, when stimulants and opiates were freely administered; and the length of time ere recovery took place, even in the most favorable instances of the malady, when these agents were given more sparingly and cautiously, long since convinced me that their tendency is highly dangerous. I do not say that I never would give a stimulant in delirium tremens. It may possibly happen, although I never have met with such a case, that in the advanced stage of the affection, the pulse may begin to falter, the heart lose its usual rhythm, the surface of the body to become of a leaden hue, the tremors to disappear, and subsultus tendinum occur, and delirium of a muttering character only continue, when I should certainly say that the flagging powers of life would require to be sustained by some diffusible stimulus. Here there would be no alternative. Then, again, I would not hesitate to give an allowance of his usual stimulus to an habitual drunkard when affected with a wound or ulcer to obtain a healthy action therein, or to administer stimuli of one kind or another freely in ordinary fever, or in the typhoid state of traumatic delirium, so that his circulation may be enabled to keep up the functions of organic life until food could be made use of. This would only be using legitimate means to maintain his ordinary condition of body; but it is quite another thing to prescribe alcohol when the individual is already manifestly in a state of alcoholic poison.

From all that I have seen and read, I believe that the combination of stimuli with opiates is a most hazardous practice in the treatment of delirium tremens; for while the former increases the determination of blood to the head, the latter is apt to occasion engorgement there, and thus, I have no doubt, they are the joint cause of many sudden deaths, and of many incurable palsies of body and mind—indeed, of the great proportion of those casualties which take place, and for which the disease, and not the treatment, is blamed.

Opium given alone in delirium tremens is, I am aware, almost universally considered by the profession to be quite an indispensable agent—the *sine qua non*—for securing what is called the critical sleep; and hence it is prescribed in smaller or larger doses in as routine a manner as sulphur is for the itch, or colchicum for gout. Notwithstanding this high estimation of its value, however, I hesitate not to say that I consider it a very doubtful remedy, even in the most promising cases of the disease, and a most dangerous one in others. It is well known that a moderate dose of opium in dilirium tremens, so far as regards its action on the brain and nervous system, is in the first instance exciting and preventive of sleep. I have frequently seen such doses as in other affections would have been considered very large, in this greatly increase the agitation and excitement after each successive administration; and although sleep was secured at times, it was but short and disturbed, and followed by delirium as violent as before. Besides, the most unmanageable cases of delirium tremens which are met with, are those affecting opium or morphine eaters, who appear to be extremely liable to this disease if they indulge in spirituous liquors. From the use of opium or morphia alone, as I have already stated, I believe that true delirium tremens never occurs; but with the unfortunate slaves of this debasing habit, a very short course of intemperance is sufficient to develope it. I have also remarked in several of these instances, that if, during the attack the usual dose of the narcotic is taken under the impression that it would soothe distress and procure sleep, more especially if that dose be morphia —which is apparently much more stimulating in this affection than opium—the paroxysm is generally aggravated. It is evident, then, that if opium is to be used at all in delirium tremens, it must be given in a large dose (in from two to three or more grains, and repeated at intervals of a few hours); and it is thus generally given, the object being to overstep the stage of excitement, and force on the desired sleep. Now the acknowledged effect of a large opiate on the encephalon is to occasion engorgement of the vessels, more especially of the veins, and consequently, the larger the dose, the greater will be the amount of sanguinous compression of the brain. What, then, must be the probable result in a disease in which there is already, if not an approach to arachnitis, at least a very excited action of the meninges, and a preternatural loading of the vessels generally? The cerebral functions are oppressed, and at length overwhelmed, and sub-arachnoid effusion is the result. The symptoms attending this untoward event are characteristic. Sleep is obtained, but it goes on deepening, and, as it becomes more profound, the pulse becomes smaller and less frequent, the surface of the body covered with a cold sweat, the face pale, the pupils contracted, the breathing slow and soft (although sometimes stertorous). An epileptic fit may now occur and terminate the scene, or the powers of life gradually become more and more depressed, and the victim perish as if in a profound and gentle sleep. Now this progress and catastrophe, although viewed as evidence of an unmanageable—a malignant form of the disease, in a bad subject, is nothing more than the common course and result of injudicious management. Even Graves, who prescribed opium in delirium tremens in the manner I will afterwards notice, warns emphatically against its premature and incautious use.

"Opium," he says, "if given in the beginning, will increase the congestion and bring on sub-arachnoid effusion. I treated a case of delirium tremens in this way too boldly, and the man died of sub-arachnoid effusion; it was a lesson to me, and I advise you to profit by my experience."

I am convinced that it is in this way very many of the sudden deaths we hear of in delirium tremens occur. I saw it frequently in early practice, and have seen it occasionally since in the practice of others; and I am persuaded that any practitioner who has been accustomed frequently to treat this affection with large doses of opium, will be able, on reflection, to explain his want of success, and the occurrence of casualties. When, in fact, recovery takes place after a long sleep forced on by a large opiate, it is simply from the wonderful conservative power of nature resisting the evil influence of the agent, just as some will recover from a severe apoplexy or palsy. The practice is one of the utmost hazard. If death were the certain alternative in delirium termens, should sleep not be early obtained—for it is said that "the patient must sleep or die"—there might be some reason in attempting to force on the sleep by opiates. This, however, is certainly not the case, and consequently such interference is not only uncalled for, but most improper, when there is danger to be apprehended from the practice. Sleep occurs as the natural, the favorable crisis, or rather termination of the disease; for it is not to be viewed as a part of the affection, or in the same light as we are accustomed to regard a critical sweat or other discharge. It is the result and a salutary relaxation succeeding a state of dangerous tension. It will take place in the mild but genuine forms of the affection at the proper period, which, as I have already remarked, is on the second or third day, when the paroxysm has run its course, when the peculiar erythism, the "nervous irritability," is brought to an end, and a condition of "exhausted nervous power" now truly produced. That this may likewise happen in severe examples of the disease, although no opiate of any kind is given, the cases with which I shall close the present paper [omitted in this Journal] will prove; and while I am convinced that the plan of treatment now to be recommended will be found the most efficacious, I have no hesitation in saying, that in a larger proportion of instances, sleep would take place spontaneously at an earlier period, and the subsequent condition of the patient be much more sound and safe, by doing nothing at all, than by the use of opiates. I have seen very decided cases of the disease recover well when a mere placebo was given with a view to keep up the appearance to friends of something being done, and prevent them from using as remedies things which would be hurtful. Dr. Ware, of Boston, in an excellent memoir on delirium tremens, strongly advocates from experience the do-nothing plan. Among other things, he says of opium:—

"In the cases which I have formerly treated with opium, and which have at last terminated well, a salutary sleep has not taken place till the close of the third day, let the quantity of opium be what it would. I have, indeed, seen sleep induced by opium at an earlier period, but it was premature, it passed into a state of coma, and the patient died. I am satisfied, therefore, that in cases of delirium tremens, the patient, so far as the paroxysm alone is concerned, should be left to the resources of his own system, particularly that no attempt should be made to force sleep by any of the remedies which are usually supposed to have that tendency, more particularly that this should not be attempted by the use of opium."

Dr. Cahill also cites several cases of the genuine disease, in which he found opiates decidedly injurious, and treatment without them salutary.

The treatment recommended by Dr. Graves, to which I have already referred, is advocated on the ground that opium is highly dangerous in the early part or the paroxysm. His rule of practice is to begin with tartar-emetic alone, with the view of combating vascular excitement, then to add a little opium, and gradually to increase the quantity, keeping its action carefully guarded and controlled by the antimony, until at last, when engorgement of the cerebral vessels is no longer to be apprehended, to use opium alone. If opium is to be given at all in delirium tremens, this is certainly the safest mode of prescription. For some time I tried it, but from considerable previous experience of the beneficial effects of antimony in this disease, I soon became convinced that it was from that agent solely, especially its effects in the first stage, the ultimate benefit was derived; that the relative quantity of opium employed at first, is too small* to counteract the power of the antimony, or to produce any notable effect whatever; that in ordinary cases, ere the time arrives for increasing much the amount of the opium, the affection has run, or nearly so, its natural course, and the period for the salutary sleep commencing is at hand; and that when a greatly-increased dose is given before this much-wished-for change has arrived, there is a proportional increase of excitement and consequent delay of its occurrence.

From these considerations, I resumed the use of antimony alone; and during the last ten years, I have treated upwards of eighty cases of the genuine disease, many of them very severe ones, with uniform success—not only in regard to the speediness of the immediate recovery, but the comparatively thorough restoration to a healthy condition of body and mind—as much so, at least, as could be expected in individuals, many of whom had been, and were likely soon again to become habitual drinkers. The dose which I have been accustomed to give, has ranged from one quarter to one half of a grain, in simple solution, every two hours, sometimes at shorter intervals, according to the degree of excitement and irritability. The action of the antimony appears to be chiefly sedative. Its direct action is to reduce the vascular excitement of the brain, soothe the nervous system, and diminish muscular power; and its more indirect action is exerted on the functions of the skin, kidneys and intestinal canal. In two or three instances only, have I found it necessary to suspend its employment, in consequence of diarrhœa and hemorrhagic discharge from the bowels; and in these cases I substituted digitalis and ipecacuan. with marked benefit; and I do not recollect of ever seeing it produce continued vomiting, although occasionally I have found the first or second dose eject from the stomach a quantity of bile. It is for the sake of its emetic effect that, in Germany and America, it has been prescribed

*Dr. Grave's formula for first use is:—R. Antimon. tart., gr. iv., tinct. opii, dr j.; aquæ, oz. viij. Signa. A tablespoonful to be taken every second hour. There is thus in each dose only five drops of laudanum to one fourth of a grain of antimony.

in large oft-repeated doses, even from four to seven grains every hour, and that too, according to report, without benefit. But although there is, doubtless, extraordinary tolerance of this agent in delirium tremens, I do not think that the use of such, or any other very heroic means, are warranted. Bleedings, large opiates, or large doses of tartar emetic, are all, although certainly not equally unsafe, and therefore to be deprecated. An antimonial course of treatment in moderation, and with the design I have indicated, gently diminishes excited action, induces weariness of muscle, general nervous exhaustion, and mental languor. It thus removes all hindrances to the occurrence of the salutary sleep. It prepares the way for it, not by forcing, but by favoring it; and when the individual, exhausted, seeks his couch, and finds repose, that goes on, not as a drugged sleep, but as a purely natural and profound repose, from which he awakes with restored reason and muscular control.

Although I have recommended the tartrate of antimony as a chief remedy in delirium tremens, there are several other means essential to its successful treatment. In the department of medicinal agents, however, I have only further to suggest, that, should the bowels not be moved by the antimony, the compound powder of jalap (ʒ. j.) will generally be found speedy and efficacious.—The other means of cure belong strictly to regimen and diet; and the first of these in importance is bodily freedom. Nothing is more hurtful in delirium tremens than the restraint, particularly that of the strait-waistcoat. I have seen instances, and heard of many more, where I have no doubt the cerebral excitment was so increased by the never-ceasing struggle for liberty, that fatal convulsions at last afforded release. All the control required is the presence of one or two judicious attendants, who will humor the patient in his whims and fancies; who will speak and act regarding them so as to assure him of safety, and to relieve him of apprehension, which is the most characteristic feature of the delirium; and who will mildly but firmly interpose, if he attempts anything which may accidentally prove injurious to himself or others. Of course, injury inflicted wrathfully or vindictively, is not to be anticipated, for rage, violence or outrage, do not occur in this remarkable disease, but only in that affection which I have already briefly noticed, and with which it is sometimes confounded, namely, the madness of drink. Hence the frequent accounts met with in the public prints, of homicidal, suicidal, and other violent acts, said to be perpetrated during fits of delirium tremens, originate in an entire misapprehension of the nature of the two diseases. The apartment, however, in which the delirium tremens patient is confined, should be well secured, for he may rush out at the door, or jump out of the window, in the fright and frenzy of supposed danger. The larger, too, the room is, the better, that he may have ample space to advance and retreat, according as he wishes to scrutinize or avoid a suspicious or distressing object of his fancy; to arrange and re-arrange articles of furniture; or to carry on, after a fashion, the duties of some bustling occupation. All this expenditure of muscular effort, without any restraint, aids greatly the antimony in producing a safe kind and amount of physical and mental exhaustion, from which the patient, languid and worn out, at last lies down voluntarily, and falls into the much-desired sleep. It is thus, too, that the "walking drill," according to Dr. Blake's experience in the West Indies, was found efficacious in warding off attacks of delirium tremens in the case of drunken soldiers; not, however, as supposed, from the exercise proving a new stimulus in place of the rum, to which they had no access, but from its wearing-out effect, while the proper nutrition of the body was maintained. No one would ever think of ordering continued and monotonous hard work, and muscular fatigue, for an affection of "exhausted nervous power."

During the entire paroxysm of the attack, it is of some consequence to afford the patient abundance of light; not, however, as supposed by Dr. Blake, for its stimulant or excitant effect, but for its aid in correcting false optical impressions. The excited brain is very apt to receive erroneous impressions, from the appearance of surrounding objects, if there is an uncertain light. Hence the exaggeration of many of those agitating and terrifying illusions and phantasms which more distinct vision would prevent or quickly dispel. During the daytime, therefore, there should be no half-closed shutters, nor half-drawn blinds or curtains, but advantage taken of the clearest light available; and during the evening or night, the more distinct the artificial light is, so much the better. Perhaps perfect darkness may serve the purpose equally well; but this can be available only in the well-padded chamber of a lunatic asylum; and besides, in private practice, the other parts of the plan of treatment here recommended, which require the presence of an attendant to regulate the doses of antimony, or other sedative, and to administer, from time to time, suitable nourishment, could not be carried on without the admission of light. This leads me to remark, in conclusion, that during the administration of the tartar-emetic, I give, at intervals of a few hours, a moderate quantity of good beef-tea, mutton broth, or chicken soup. Thus, while the vascular action in the brain is being subdued, and the nervous system liberated from the presence of the alcoholic poison, the functions of organic life are sustained, and a better ultimate recovery is secured.—*Edin. Jour. Med. Science.*

